# Yam Daabo interventions’ effects on postpartum family planning use in Burkina Faso at 24 months after childbirth

**DOI:** 10.1186/s12889-021-10964-w

**Published:** 2021-05-19

**Authors:** Abou Coulibaly, Adama Baguiya, Franck Garanet, Nguyen Toan Tran, Tieba Millogo, Wambi Maurice Evariste Yaméogo, Ivlabèhirè Bertrand Meda, Blandine Thieba, Séni Kouanda

**Affiliations:** 1 Unité de Surveillance Démographique et de Santé (Kaya-HDSS), Institut de Recherche en Sciences de la Santé (IRSS), 03 B.P. 7047, Ouagadougou, 03 Burkina Faso; 2 Ecole doctorale Sciences de la Santé, Université Joseph KI-ZERBO, 03 B.P. 7021, Ouagadougou, 03 Burkina Faso; 3 Faculty of Medicine, University of Geneva, Geneva, Switzerland; 4 Australian Centre for Public and Population Health Research, Faculty of Health, University of Technology, PO Box 123, Sydney, NSW 2007 Australia; 5 Institut Africain de Santé Publique, 12 B.P, Ouagadougou, 199 Burkina Faso; 6 Institut de Recherche en Sciences de la Santé (IRSS), 03 B.P. 7047, Ouagadougou, 03 Burkina Faso; 7 Unité de formation et de recherche en sciences de la santé, Université Joseph KI-ZERBO, 03 B.P. 7021, Ouagadougou, 03 Burkina Faso

**Keywords:** Family planning, Interventions, Use, Long-term effects, Postpartum

## Abstract

**Introduction:**

After testing the interventions for improving the prevalence of contraceptive use, very few studies have measured the long-term effects thereafter the end of the implementation. This study aimed to measure *Yam Daabo* interventions’ effects on contraceptive use in Burkina Faso at twelve months after completion of the intervention.

**Methods:**

Yam Daabo was a two-group, multi-intervention, single-blind, cluster randomized controlled trial. Interventions comprised refresher training for the provider, a counseling tool, supportive supervision, availability of contraceptive services 7 days a week, client appointment cards, and invitation letters for partners. We used generalized linear mixed-effects models (log Poisson) to compare the modern contraceptive prevalence at 12 months post-intervention in the two groups. We collected data between September and November 2018. We conducted an intention-to-treat analysis and adjusted the prevalence ratios on cluster effects and unbalanced baseline characteristics.

**Results:**

Twelve months after the completion of the Yam Daabo trial, we interviewed 87.4% (485 out of 555 women with available data at 12 months, that is, 247/276 in the intervention group (89.5%) and 238/279 in the control group (85.3%). No difference was observed in the use of hormonal contraceptive methods between the intervention and control groups (adjusted prevalence ratio = 1.21; 95% confidence interval [CI] = [0.91–1.61], *p* = 0.191). By contrast, women in the intervention group were more likely to use long-acting reversible contraceptives (LARC) than those in the control group (adjusted prevalence ratio = 1.35; 95% CI = [1.08–1.69], *p* = 0.008).

**Conclusion:**

Twelve months after completion of the intervention, we found no significant difference in hormonal contraceptive use between women in the intervention and their control group counterparts. However, women in the intervention group were significantly more likely to use long-acting reversible contraceptives than those in the control group.

**Trial registration:**

The trial registration number at the Pan African Clinical Trials Registry is PACTR201609001784334. The date of the first registration is 27/09/2016.

**Supplementary Information:**

The online version contains supplementary material available at 10.1186/s12889-021-10964-w.

## Introduction

Literature shows that several interventions have been tested to improve the prevalence of contraceptive use [1, 2]. In 2015, we tested a package of interventions in two African countries, Burkina Faso and the Democratic Republic of the Congo, in a randomized cluster trial design called Yam Daabo [3, 4]. The postpartum family planning interventions package had two types of interventions. The first one comprised three facility-oriented interventions: refresher training of service providers, regularly scheduled and strengthened supportive supervision of providers, enhanced availability of services 7 days a week. The second had three individual-based interventions: a postpartum family planning counseling tool, appointment cards for women, and invitation letters for partners. The conception of the new counseling tool takes into account all new World Health Organization (WHO) recommendations on family planning service provision. The package was designed through participatory action research, and the process and contents are detailed elsewhere [4]. After 12 months of follow-up (which marks the end of the interventions), the main results showed a significant increase in the use of appropriate modern contraceptive methods among women receiving the interventions compared to women not receiving the interventions. In Burkina Faso, at 12 months, the modern contraceptive prevalence rate was 55% among women in the intervention group and 29% among women in the control group (adjusted prevalence ratio: 1.79, 95% CI = 1.30–2.47) [5]. In the Democratic Republic of the Congo, at 12 months, 46% of the women in the intervention group and 35% of the women in the control group were using modern contraceptives (adjusted prevalence ratio: 1.58, 95% CI = 0.74–3.38), with significant differences in the use of contraceptive implants (22% vs. 6%; adjusted prevalence ratio: 4.36, 95% CI = 1.96–9.70) [6].

However, many questions remain unanswered a few months after these types of interventions because most studies examine the effects of interventions immediately after the interventions’ completion. So, very few studies have measured the long-term effects of the interventions that were effective after they were discontinued.

As three (out of six) of Yam Daabo’s interventions were individual-based, we hypothesized that (i) health workers could continue to offer some elements of the intervention package to women visiting the health centers, (ii) the effects may persist even after the interventions were stopped since women could continue to receive counseling during and after the first year postpartum, (iii) women in the intervention group not using the methods at the end of the interventions because of past and current exposures could start using contraceptive methods one year after giving birth. We hypothesize that appropriate contraceptive methods use would remain high at 12 months post-intervention among women in the intervention group compared to those in the control group. Therefore, this study aimed to measure the effects of the Yam Daabo trial interventions on contraceptive methods use in Burkina Faso, 12 months after the end of the interventions.

## Methods

We conducted an evaluation of the Yam Daabo trial’s effects at twelve months after intervention completion. The intervention was implemented in the Yako health district (Centre-North region, Burkina Faso) between July 27, 2016 and February, 28, 2018.

Burkina Faso is a sub-Saharan African country, with an estimated population of 20,321,000 in 2019 [7]. According to Blumenberg et al., the modern contraceptive prevalence rate was 31.9% in December 2018, based on the analysis of Performance Monitoring and Accountability 2020 (PMA2020) data [8]. Ahmed et al., using the Family planning 2020 data, reported an estimated unmet need for modern contraception of 27.2% in 2017 [9]. As in most countries in sub-Saharan Africa, cultural norms favor large families, with higher fertility rates in rural areas than in urban areas. In 2019, Burkina Faso had 5.6 live births per woman [7].

### Description of the yam Daabo trial

The Yam Daabo intervention was a complex, multicentre, randomized cluster trial implemented in two countries, Burkina Faso and the Democratic Republic of the Congo. Methodological details on the *Yam Daabo* are available in the published protocol [3]. We randomized each country’s health centers into two groups during the intervention phase: intervention and control. The health centers in the experimental group offered six postpartum family planning interventions that were identified as solutions to the barriers identified during the formative phase of the project and are already described in another article [4]. The health centers in the control group offered the usual postpartum family planning care. We included and followed women at the third trimester of their pregnancy over 12 months after delivery. As said above, Yam Daabo’s interventions comprised refresher training for the provider, counseling tool, supportive supervision, availability of contraceptive services 7 days a week, client appointment cards, and invitation letters for partners. Participants received individual-based interventions during their pregnancy and during the postpartum period, according to national practice (typically on clinical discharge [24–48 h], at 6 days, 6 weeks, then at months 6 and 9, before the trial exit at month 12 postpartum). All pregnant women were eligible to participate in the study if (1) they were in their third pregnancy trimester; (2) the status of the pregnancy and the woman allowed for a birth at the health center; (3) the woman had the intention to attend antenatal care, delivery, and postnatal care at the health center; (4) the woman did not participate in another study; and (5) we obtained informed consent. Research assistants collected data on paper-based case report forms. The WHO team in Geneva developed the case report forms with inputs from the country’s research teams. Each health center of the cluster randomized controlled trial had a research assistant who was trained to adhere to the study manual and standard operating procedures for data management, which are common to both study countries and have been developed by WHO. In each country, the eight sites were matched by pairs according (1) the average number of deliveries per month, (2) the ratio of health workers per population, and (3) the settings (rural, urban). Within each pair, we randomly selected the site assigned to the experimental intervention. This randomization was done four times. No restriction in the randomization process was required. All consecutive and eligible participants were included in the clusters. Due to the nature of the interventions, participants, health staff, research assistants assigned to each centre, and the rest of the research team members could not be masked to the cluster assignments.

The trial was approved by the WHO Ethics Committee and the Health Research Ethics Committee of Burkina Faso and has been registered in the Pan-African Clinical Trials Registry (registration number: PACTR201609001784334 on 27/09/2016).

No interim analysis was conducted during this study. At the end of the 12-month follow-up, analyses on contraceptive use at each of the contact points (day 6, week 6, month 6, and month 12) were conducted. These results for Burkina Faso and the Democratic Republic of the Congo have already been published [5, 6].

### Type of study

We conducted a randomized, clustered, controlled trial extending the follow-up of women to 12 months post-intervention.

### Population

Our study population was all women who completed *Yam Daabo* trial in Burkina Faso at 12 months and those who were not found because of travel. We included women lost to follow-up at 12 months because they were all exposed to the interventions during pregnancy, delivery, and the postpartum period.

However, we did not include in this study women who were pregnant at the 12th month, those who withdrew from the study and finally women who died before the 12th month.

### Additional collection period

We collected data from September 15, 2018, to November 15, 2018.

### Key measures

The primary outcome of this study was the contraceptive method use. We used the classification adopted by the WHO in 2015 [10]. Thus, for this analysis, the groups of methods were defined as follows:

- Long-acting reversible contraceptive, including implants and intrauterine devices
- Short-acting contraceptive methods, including injectables, pills, emergency contraception, male and female condoms
- Permanent methods (male and female sterilization)
- The lactational amenorrhea method

Next, we also classified the contraceptive methods into “modern and appropriate methods” and “non-modern or inappropriate methods.” The non-modern methods comprised traditional methods, withdrawal, and abstinence. Inappropriate methods include lactational amenorrhea (if used after 6 months) and calendar-based methods (if used during the first 12 months postpartum).

The main independent variable was exposure to interventions, defined as the binary variable (coded 1 for the intervention group and 0 for the control group).

Moreover, we measured the frequency of pregnancies in both groups of women.

### Data collection at 12 months post-intervention

Five interviewers collected the data (They were part of the interviewing team that collected follow-up data up to 12 months postpartum). To locate the target participants, they used the women’s identification documents, which contained their telephone numbers. Maternity health workers and community-based health workers provided valuable assistance in the search for women. They conducted interviews mainly in health centers and women’s homes or other secure public places chosen by the woman beforehand. Data on contraception use were extracted into the health centers registers.

However, the 12-months post-intervention study was conducted only in Burkina Faso not in the Democratic Republic of the Congo. This data collection was decided by the Burkina research team to measure family planning use after the interventions had ended.

### Data processing and statistical analysis

EpiData and Stata 15.1 software were used for data entry and analysis, respectively. To ensure the comparability of results with published data on intervention effectiveness at 12 months postpartum, we used generalized linear mixed-effects (log Poisson) models were used to measure the effects of interventions on contraceptive use with a significance threshold of 5%. We adjusted the measure of association on sociodemographic variables unequally distributed between the intervention and control groups, at inclusion. We also corrected for a possible cluster effect by taking into account any correlation that might exist.

We followed Consolidated Standards of Reporting Trials extension for pragmatic trials guidelines to write the manuscript (supplementary file 1).

## Results

**Fig. 1:**
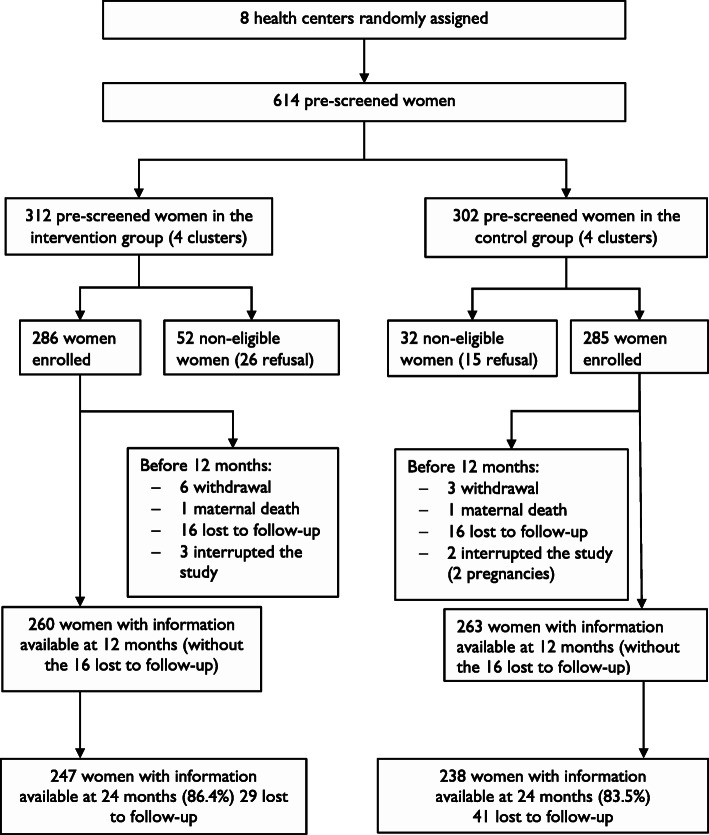
Flow diagram

Twelve months after the end of the *Yam Daabo* interventions, we interviewed 485 (87.4%) of 555 women (those with available data at 12 months or lost to follow-up). The lost to follow-up rates were 10.5% in the intervention group (247 of 276 women included) and 14.7% in the control group (238 of 279 women included). The flow diagram, presented in Fig. 1, summarizes this information. However, no statistical difference was observed in the lost to follow-up rates between the two groups (*p* = 0.1362).

The chi-square test comparing women’s sociodemographic and gynecological characteristics at baseline showed no significant difference in any of the variables except the total number of abortions (*p* = 0.01). These data are presented in Table 1.

**Table 1:** Baseline characteristics of women who were interviewed at 12 months post-intervention

	Intervention ***n*** = 238 n(%)	Control ***n*** = 247 n(%)
Women’s age		
Under 20	31 (13.0)	46 (18.6)
20–29	123 (51.7)	133 (53.8)
30 and more	84 (35.3)	68 (27.5)
Number of pregnancies		
None	36 (15.1)	47 (19.0)
1–3	106 (44.5)	123 (49.8)
4–6	77 (32.4)	63 (25.5)
≥ 7	19 (8.0)	14 (5.7)
Number of living children		
None	42 (17.6)	53 (21.5)
1–3	121 (50.8)	131 (53.0)
≥ 4	75 (31.5)	63 (25.5)
Number of previous live births		
None	42 (17.6)	49 (19.8)
1–3	112 (47.1)	126 (51.0)
≥ 4	84 (35.3)	72 (29.1)
Number of stillbirths		
None	224 (94.1)	222 (89.9)
≥ 1 stillbirth	14 (5.9)	25 (10.1)
Number of abortion*		
None	206 (86.6)	231 (93.5)
≥ 1 abortion	32 (13.4)	16 (6.5)
Education status		
No education	163 (68.5)	174 (70.4)
Primary school	35 (14.7)	36 (14.6)
Secondary/Tertiary	40 (16.8)	37 (15.0)
Marital status		
Not in union	2 (0.8)	4 (1.6)
In union	236 (99.2)	243 (98.4)
Occupation		
No occupation	1 (0.4)	0 (0.0)
Student	24 (10.1)	19 (7.7)
Housewife/Farmer	185 (77.7)	201 (81.4)
Salaried employee	2 (0.8)	5 (2.0)
Tradeswoman	26 (10.9)	22 (8.9)
Family planning use prior to the trial		
No	161 (67.6)	178 (72.1)
Yes	77 (32.4)	69 (27.9)
Current pregnancy planned		
Yes	115 (48.3)	113 (45.7)
No	123 (51.7)	134 (54.3)

*: significant difference (*p* = 0,01)

Results showed no difference in modern contraceptive use between the intervention and control groups at 12 months post-intervention (58.0% vs. 47.4%; Adjusted prevalence ratio = 1.21; 95% CI = 0.91–1.61, *p* = 0.191). The use of long-acting reversible contraceptives was higher among women in the intervention group than in those in the control group with a statistically significant difference (37.4% vs. 27.5%; Adjusted prevalence ratio = 1.35; 95% CI = 1.08–1.69, *p* = 0.007). No significant difference was noted in the use of short-acting methods (20.6% vs. 19.8%; Adjusted prevalence ratio = 1.00, 95% CI = 0.55–1.81, *p* = 0.992). Among the non-modern or inappropriate methods, only one woman was using the standard days’ method. None of the women used a permanent method (vasectomy or tubal ligation) at 12 months post-intervention. Table 2 expressed these results.

**Table 2:** Contraception use at 12 months post-intervention

	Intervention group n/N	Control group n/N	p	Adjusted prevalence ratio (95%CI)^a^
Modern and appropriate methods	138/238 (58.0%)	117/247 (47.4%)	0.191	1.21 (0.91–1.61)
Long-acting methods	89/238 (37.4%)	68/247 (27.5%)	0.007	1.35 (1.08–1.69)
Short-acting methods	49/238 (20.6%)	49/247 (19.8%)	0.992	1.00 (0.55–1.81)
Non-modern or non-appropriate methods	1/238 (0.4%)	0/247 (0%)	–	–
No contraceptive method	100/238 (42%)	130/247 (53%)	0.224	0.8 (0.56–1.15)

^a^: adjusted results on the number of abortions

Concerning the frequency of pregnancies, we noted no significant difference between women in the intervention and control groups (21 out of 238 pregnant women in the intervention group versus 23 out of 247 in the control group, 8.8 and 9.3%, respectively).

## Discussion

This analysis has two main findings. First, *Yam Daabo* trial interventions’ effects on modern family planning use persisted 12 months after the interventions ended, but no statistically significant difference was noted between the experimental and control groups. Second, a statistically significant difference was noted in its effects on long-acting reversible contraceptives use.

Some authors, including Tu et al. [11], Speizer et al. [12, 13], Subramanian et al. [14], and Jejeebhoy et al. [15], after evaluating the long-term effects of certain interventions related to contraceptives’ provision several months after the cessation of interventions noted that the effects varied in terms of the magnitude. Indeed, in Shanghai, Tu et al. noted a significant difference in contraceptive method use and reported a higher contraceptive use in the group of young people who benefited from the interventions 28 months after the cessation of interventions [11]. Subramanian et al. [14] and Jejeebhoy et al. [15] reported the persistence of effects 4 to 8 years after the end of the Pathfinder International’s Promoting Change in Reproductive Behavior project, implemented between 2001 and 2012 in Bihar (India), on contraceptive use among young married couples. Speizer et al. noted the persistent effects of a community-based program on contraceptive use in two cities where the program had been implemented [12] and a change in the effects of the same program on the quality of family planning services after the program ended [13]. The interventions’ nature can explain the durability of *Yam Daabo* interventions effects (significant difference in the use of long-acting reversible contraceptives) with three individual-based rather than community-based. One could be led to believe that the skills women learned during the implementation phase of the interventions guided them for 12 months after the interventions’ completion. This is a cause for satisfaction because the cessation of the interventions coincided with the end of repeated contact period between postpartum women and health workers. In Burkina Faso’s context, these contacts are limited to 12 months postpartum.

The non-significant difference at 24-month in modern contraceptive use between the experimental and control groups could be explained by the effects of two campaigns of free contraceptive methods’ distribution since the last follow-up. These two campaigns were conducted in all public health facilities (7 days each, including one campaign in November and another one in June each year). Compared with the results published at 12 months, this non-significant difference in the modern contraceptive prevalence can be explained by the increased contraceptive use in the control group (29% at 12 months [5] vs. 47.4% at 24 months). This net increase in the control group could be related to the effects of the national family planning week, which is celebrated every June and November and covers all regions of Burkina Faso, during which contraceptives are provided free of charge. Such a campaign for free contraceptive methods could therefore have more effect in the control group (which included women who used the methods less) than in the intervention group since there was a 55% use rate at 12 months [5]. It could be argued that because of the higher proportion of unmet needs in the control group, it is easier to register new users as soon as free distribution campaigns take place. Current data on the modern contraceptive prevalence in the general population could help us better comprehend this rate’s evolution in the control group. Thus, if contraceptive methods were free, women would use more modern methods. In the Burkinabe context, the implementation of the interventions tested in this study, combined with the free distribution of contraceptive products (already ongoing in Burkina Faso since 2019 in some regions and since 2020 throughout the country), could allow the widespread use of modern contraceptive methods.

Our study had several limitations. First, the proportion of people who were lost to follow-up was slightly high. However, this did not affect our results’ internal validity in terms of power since the initial hypothesis was that 60 women per health center, 480 women in total, should be included to achieve the minimum desired power [3]. However, we selected and surveyed 485 women. Another limitation was that we had no contact with the women 12 months after the study. As a result, the participants may have forgotten several episodes.

## Conclusion

This study showed that the effects of interventions in improving contraceptive use can be sustained for up to 12 months after the interventions are completed. Indeed, women continued to use appropriate contraceptive methods and long-acting reversible contraceptives 12 months after the interventions were discontinued. The modern contraceptive prevalence level of the intervention group may have important implications in Burkina Faso. Indeed, if *Yam Daabo’s* realistic interventions are implemented and combined with free contraceptive methods, nearly 3 out of 5 women will still be using modern contraceptive methods at 24 months. This result is more significant as contraceptive methods have been provided free of charge in Burkina Faso’s public health facilities since 2020.

## Supplementary Information

**Additional file 1.** Related file 1: Checklist of items for reporting pragmatic trials.

## Data Availability

The datasets used and/or analysed during the current study are available from the corresponding author on reasonable request.
